# Obesity Alters the Microbial Community Profile in Korean Adolescents

**DOI:** 10.1371/journal.pone.0134333

**Published:** 2015-07-31

**Authors:** Hae-Jin Hu, Sin-Gi Park, Han Byul Jang, Min-Gyu Choi, Kyung-Hee Park, Jae Heon Kang, Sang Ick Park, Hye-Ja Lee, Seung-Hak Cho

**Affiliations:** 1 Division of Enteric Diseases, Center for Infectious Diseases, Korea National Institute of Health, Heungdeok-Gu, Cheongju, Republic of Korea; 2 Center for Biomedical Science, Korea National Institute of Health, Cheongju, Chungcheongbuk-do, Republic of Korea; 3 TheragenEtex Bio Institute Inc., Suwon, Republic of Korea; 4 Department of Family Medicine, Hallym University Sacred Heart Hospital, Hallym University, Gangnam, Seoul, Republic of Korea; 5 Department of Family Medicine, Hallym University Sacred Heart Hospital, Hallym University, Anyang, Gyeonggi-do, Republic of Korea; 6 Department of Family Medicine, Obesity Research Institute, Seoul Paik Hospital, College of Medicine, Inje University, Seoul, Republic of Korea; Wageningen University, NETHERLANDS

## Abstract

Obesity is an increasing public health concern worldwide. According to the latest Organization for Economic Co-operation and Development (OECD) report (2014), the incidence of child obesity in Korea has exceeded the OECD average. To better understand and control this condition, the present study examined the composition of the gut microbial community in normal and obese adolescents. Fecal samples were collected from 67 obese (body mass index [BMI] ≥ 30 kg/m^2^, or ≥ 99^th^ BMI percentile) and 67 normal (BMI < 25 kg/m^2^ or < 85^th^ BMI percentile) Korean adolescents aged 13–16 years and subjected to 16S rRNA gene sequencing. Analysis of bacterial composition according to taxonomic rank (genus, family, and phylum) revealed marked differences in the *Bacteroides* and *Prevotella* populations in normal and obese samples (*p* < 0.005) at the genus and family levels; however, there was no difference in the *Firmicutes*-to-*Bacteroidetes* (F/B) ratio between normal and obese adolescents samples at the phylum level (F/B normal = 0.50 ± 0.53; F/B obese = 0.56 ± 0.86; *p* = 0.384). Statistical analysis revealed a significant association between the compositions of several bacterial taxa and child obesity. Among these, *Bacteroides* and *Prevotella* showed the most significant association with BMI (p < 0.0001 and 0.0001, respectively). We also found that the composition of *Bacteroides* was negatively associated with triglycerides (TG), total cholesterol, and high-sensitive C-reactive protein (hs-crp) (*p* = 0.0049, 0.0023, and 0.0038, respectively) levels, whereas that of *Prevotella* was positively associated with TG and hs-crp levels (*p* = 0.0394 and 0.0150, respectively). We then applied the association rule mining algorithm to generate “rules” to identify the association between the populations of multiple bacterial taxa and obesity; these rules were able to discriminate obese from normal states. Therefore, the present study describes a systemic approach to identify the association between bacterial populations in the gut and childhood obesity.

## Introduction

Obesity is a public health issue worldwide and tops the public health agenda in both industrialized and developing countries. Although obesity rates in Korean adults are one of lowest rates among Organization for Economic Co-operation and Development (OECD) countries (OECD report 2014, http://www.oecd.org/els/health-systems/Obesity-Update-2014.pdf), levels have increased steadily in recent decades. Notably, the rate of childhood obesity in Korea exceeded the OECD average of 23%; indeed, 25% of Korean boys (aged 5 to 17 years) are obese. The obesity rate for Korean girls (20%) is slightly below the OECD average (21%). When many of these children reach young adulthood, they suffer several obesity-related conditions, such as diabetes, heart disease, or certain types of cancer [1, 2], which places a heavy burden on the healthcare system and society in general.

Therefore, to better understand and control this epidemic, a number of studies have attempted to identify genetic and/or environmental factors associated with obesity [3–6]. The recently emerged field of metagenomics has allowed researchers to examine the microorganisms that inhabit the human gut, known as the microbiota, as a novel environmental factor associated with obesity [7–12]. These studies identified marked changes in the composition of the gut microbiota and its metabolic function in obese subjects. They also suggested that the gut microbiota plays a significant role in harvesting energy from food and storing it within the host [7]. Other studies report that the composition of the gut microbiota differs according to age and geographical location [13–16]. Taken together, these results imply that there is a marked difference in the composition of obesity-associated gut microbiota between children and adults and among populations. To date, few studies have examined the association between childhood obesity and the composition of the gut microbiota [17–19]. Studies suggesting that the *Firmicutes*-to-*Bacteroidetes* (F/B) ratio plays a role in human obesity are rather contradictory. Two studies, which were conducted in Spanish [19] and Belgian children [18], reported an increased F/B ratio; however, a study by Karlsson et al. [17] found no significant difference in this ratio in obese and lean Swedish preschool children. Recently, a metagenomic analysis of samples from European populations indicated that the number of gut microbial genes, and thus the “richness” of gut bacterial population, differed between obese and lean groups [20].

Therefore, we performed an in-depth analysis of the gut microbiota in 67 obese and 67 normal Korean adolescents aged 13 to 16 years. Sequencing the 16S rRNA genes obtained from fecal samples was performed to obtain an overall picture of the gut bacterial composition of the two groups according to taxonomic rank. Next, we performed statistical analyses to identify bacterial taxa significantly associated with childhood obesity. We then examined the correlation between microbial composition and BMI or the levels of biochemical markers. Finally, we attempted to generate rules (patterns) that associate multiple bacterial taxa with obesity, thereby allowing discrimination between obese and normal states.

## Materials and Methods

### Study participants

This study subjects comprised 67 obese adolescents (BMI ≥ 30 kg/m^2^ or ≥ 99^th^ BMI percentile) and 67 normal adolescents (BMI < 25 kg/m^2^ or < 85^th^ BMI percentile). A total of 134 adolescents aged 13 to 16 years were recruited from Seoul and Kyunggi province as part of the Korean Children and Adolescent Study (KoCAS) in 2012, which has monitored this cohort on an annual basis since they entered elementary school in 2005 aged 7 years. None of the subjects had taken antibiotics in the 4 weeks before the sampling dates and none had significant co-morbidities such as acute infection or chronic disease. The study was approved by the Institutional Review Board of the Korea Center for Disease Control and Prevention and by the Ethics Committee of Seoul Paik Hospital, Inje University (IIT-2012-092). Written informed parental consent was obtained prior to enrolment.

### Biochemical analyses

A vacutainer tube was used to collect blood samples from the antecubital vein between 9:00 AM and 11:00 AM after a 12-hour overnight fast. Within 30 minutes, plasma and serum were separated and stored at -80°C prior to further analysis. The levels of triglycerides (TG), total cholesterol (T_chol_), high-density lipoprotein-cholesterol (HDL-C), hs-crp, and glucose were measured using an autoanalyzer (model 7600II; Hitachi, Tokyo, Japan).

### Stool sampling and DNA extraction

Samples were taken by each participant at home. A fresh stool sample (~ 30 ml) was placed into a collection container with dry ice and brought to the study center within 12 h. The sample was stored at -70°C in the laboratory prior to DNA extraction. DNA was extracted using a QIAamp DNA Stool Kit (Qiagen, Valencia, CA), according to the manufacturers protocol. The quality and concentration of the DNA were checked using a Nanodrop 2000 spectrophotometer (Nanodrop Technologies, Wilmington, DE).

### Pyrosequencing of 16S rRNA

The 16S rRNA gene fragments were amplified from the extracted DNA. The following barcoded primers were designed to target the hyper-variable regions (V1 to V3) within the 16S rRNA gene: 9F (5’-CCTATCCCCTGTGTGCCTTGGCAGTC-TCAG-AC-AGAGTTTGATCMTGGCTCAG-3’ [bacteria 16rRNA primer included 90%]; 5’-CCTATCCCCTGTGTGCCTTGGCAGTC-TCAG-AC-GGGTTCGATTCTGGCTCAG-3’ [*Bifidobacterium* 16 rRNA primer included 10%]; the target region primers are underlined) and 541R (5’-CCATCTCATCCCTGCGTGTCTCCGAC-TCAG-X-AC-ATTACCGCGGCTGCTGG-3’; ‘X’ denotes the unique barcode for each subject) (http://www.ebi.ac.uk/arrayexpress/experiments/E-GEOD-61493/protocols/). PCR was performed as follows: initial denaturation at 95°C for 5 min, followed by 30 cycles of denaturation at 95°C for 30 sec, primer annealing at 55°C for 30 sec, and extension at 72°C for 30 sec, with a final elongation at 72°C for 5 min. Each sample was subjected to PCR on three separate occasions.

The quality of the PCR product was confirmed by running a sample in 2% agarose gels followed by visualization using the Gel Doc system (BioRad, Hercules, CA, USA). The amplified products were purified with the QIAquick PCR purification kit (Qiagen, Valencia, CA, USA) and equal amounts pooled. Short DNA fragments (non-target products) were removed using an Ampure beads kit (Agencourt Bioscience, MA, USA). The quality and size of the products were assessed using a Bioanalyzer 2100 (Agilent, Palo Alto, CA, USA) and a DNA 7500 chip. Mixed amplicons were used for emulsion PCR and deposited on Picotiter plates. Sequencing was performed with the GS Junior Sequencing system (Roche, Branford, CT, USA) according to the manufacturer’s instructions.

### Analysis of pyrosequencing data

Pyrosequencing data was analyzed as previously described [21–23]. Reads were sorted using unique barcodes for each PCR product. The barcode, linker, and primer sequences were then removed from the original sequencing reads. Any reads containing two or more ambiguous nucleotides, those with a low-quality score (average score < 25), or reads shorter than 300 bp, were filtered out. Potential chimeric sequences were detected using the Bellerophon method [24].

### Determination of operational taxonomic units and taxonomic classification

The pre-processed reads from each sample were used to calculate the number of operational taxonomic units (OTUs). The number of OTUs was determined by clustering the sequences from each sample using a 97% sequence identity cut-off [16, 25, 26] using QIIME software (v.1.8.0). Taxonomic abundance was counted with RDP Classifier v1.1 using a confidence threshold of 0.8 derived from the pre-processed reads for each sample. The microbial composition was normalized using the value calculated from the taxonomy abundance count divided by the number of pre-processed reads for each sample.

### Association rule mining

The CPAR (Classification based on Predictive Association Rules) algorithm was used to generate a complete rule set [27]. This algorithm is more suited to bioinformatics applications than the traditional support-confidence based measure for market basket data analysis because it uses information metrics to generate rules [28]. The CPAR algorithm was implemented by the LUCS-KDD research group (http://cgi.csc.liv.ac.uk/~frans/KDD/Software/FOIL_PRM_CPAR/foilPrmCpar.html).

The accuracy of the rules generated by CPAR is presented in terms of Laplace accuracy. Using rule *r*, Laplace accuracy is defined as follows:

$$Laplace\;accuracy\;\left(r\right)=\frac{\left(N_{g}+1\right)}{\left(N_{total}+\;m\right)}$$ (1)

where *m* is the number of target groups and *N*_*total*_ is the total number of examples that satisfy the body of the rules (total number of As in the rule, A → B). *N*_*g*_ is the number of examples that belong to the predicted target group g.

### Statistical analyses

Statistical analysis was performed with IBM SPSS version 17.0 (SPSS Inc., Chicago, IL, USA) and the R package (version 2.15.3). Variables were examined for normality, and those that were not normally distributed were log-transformed before analysis. To measure the alpha diversity of each sample, the OTUs were analyzed using the Shannon index, $H^{\prime }\text{ = - }\sum_{i=1}^{S}\left(p_{i}\text{ln}\left(p_{i}\right)\right)$ [29]. To measure beta diversity, the difference in organism composition was measured according to Bray-Curtis distance, $BC_{ij}=\;\frac{S_{i}+S_{j}–\;2C_{ij}\;}{S_{i}+S_{j}}$. Principal component analysis (PCA) was then performed using the measured beta diversities [30]. The Chi-square test was used to test whether gender showed an equal distribution between normal and obese groups. Age, height, weight, BMI, and blood profiles (all mean values) were compared using Student’s *t*-test. The association between microbiota composition and BMI or levels of biochemical markers was expressed in terms of the Pearson partial correlation coefficient (controlled for age and gender). The Kolmogorov-Smirnov test with Lilliefors significance correction was used to examine the normality of data used to test the microbial composition between obese and normal groups. Accordingly, the non-parametric Mann-Whitney U test was used for the comparison. The association between microbiota composition and BMI or biochemical markers was expressed in terms of the *Pearson’s* correlation coefficient after adjusting for age and gender. FDR multiple test correction was performed with the R package. An adjusted P-value < 0.05 was considered significant.

## Results

### Subject characteristics

The general characteristics of the study subjects/samples are shown in Table 1. The 134 participants were classified as obese or normal (67 obese and 67 normal) according to BMI status. Gender and age showed a similar distribution between the two groups; however, the individuals in the obese group were slightly taller than those in the normal group. There were significant differences in BMI, BMI z-score and the level of biochemical markers (TG, Tchol, HDLc, and hs-crp) between the two groups (Table 1). These levels of biochemical markers were significantly lower in the normal group than in the obese group.

**Table 1:** Demographic characteristics of the study participants.

	Normal	Obese	*P*
Gender (number of samples, %)			
Male	37 (55.2)	41 (61.2)	0.4836*
Female	30 (44.8)	26 (38.8)	
Age (years)	13.8 ± 0.3	14.0 ± 0.8	0.0804^†^
Height (kg)	162.9 ± 7.4	166.3 ± 7.3	**0.0101**
Weight (kg)	53.7 ± 7.8	98.2 ± 13.7	**<0.0001**
Body mass index (BMI, kg/m^2^)	20.2 ± 2.1	35.4 ± 2.9	**<0.0001**
BMI z-score	0.0 ± 0.7	2.9 ± 0.4	**<0.0001**
**Blood profiles**	N = 64	N = 68	
Triglycerides (mg/dL)	72.0 ± 40.2	143.5 ± 74.8	**<0.0001**
Total cholesterol (mg/dL)	155.2 ± 25.1	179.6 ± 27.7	**<0.0001**
HDL-cholesterol (mg/dL)	54.7 ± 7.5	44.2 ± 8.5	**<0.0001**
hs-crp (mg/dL)	0.09 ± 0.13	0.33 ± 0.36	**<0.0001**

BMI z-score = Z-score-converted value from the 2007 Korean growth chart.

Data are expressed as the mean ± SD or as a number (%).

*Chi-square test.

^†^Student’s *t*-test was used to compare the mean values for age, height, weight, BMI, and blood profiles. Bold letters signify *p* < 0.05.

### Microbial diversity across all samples

After filtering out primer sequences and low-quality and chimeric sequences, we obtained a total of 1,185,358 high-quality sequences (reads) (range, 2,065–42,522 reads) from 134 samples (A of Figure A in S1 File). Each sample was covered by an average of 8,846 reads, which is similar to the number of average reads (8,427 reads) reported in a study examining the gut microbiota of 20 Korean individuals [16]. Based on these reads, we calculated the number of OTUs to examine the diversity of the gut microbiota. The mean number of OTUs was 356 ± 140 (range, 105–876) (B of Figure A in S1 File). There was no significant difference in the number of OTUs between normal and obese individuals (Mann-Whitney U test, *p* = 0.072). In addition, we found no obvious difference in the alpha diversity (Shannon Index) values between normal and obese samples (mean number of OTUs: normal 6.94 ± 0.49; obese 6.98 ± 0.59) (Figure B in S1 File). Also, the PCA result for beta diversity did not show a differential pattern between normal and obese samples (Figure C in S1 File).

### Comparison of fecal microbial composition

**Fig 1:**
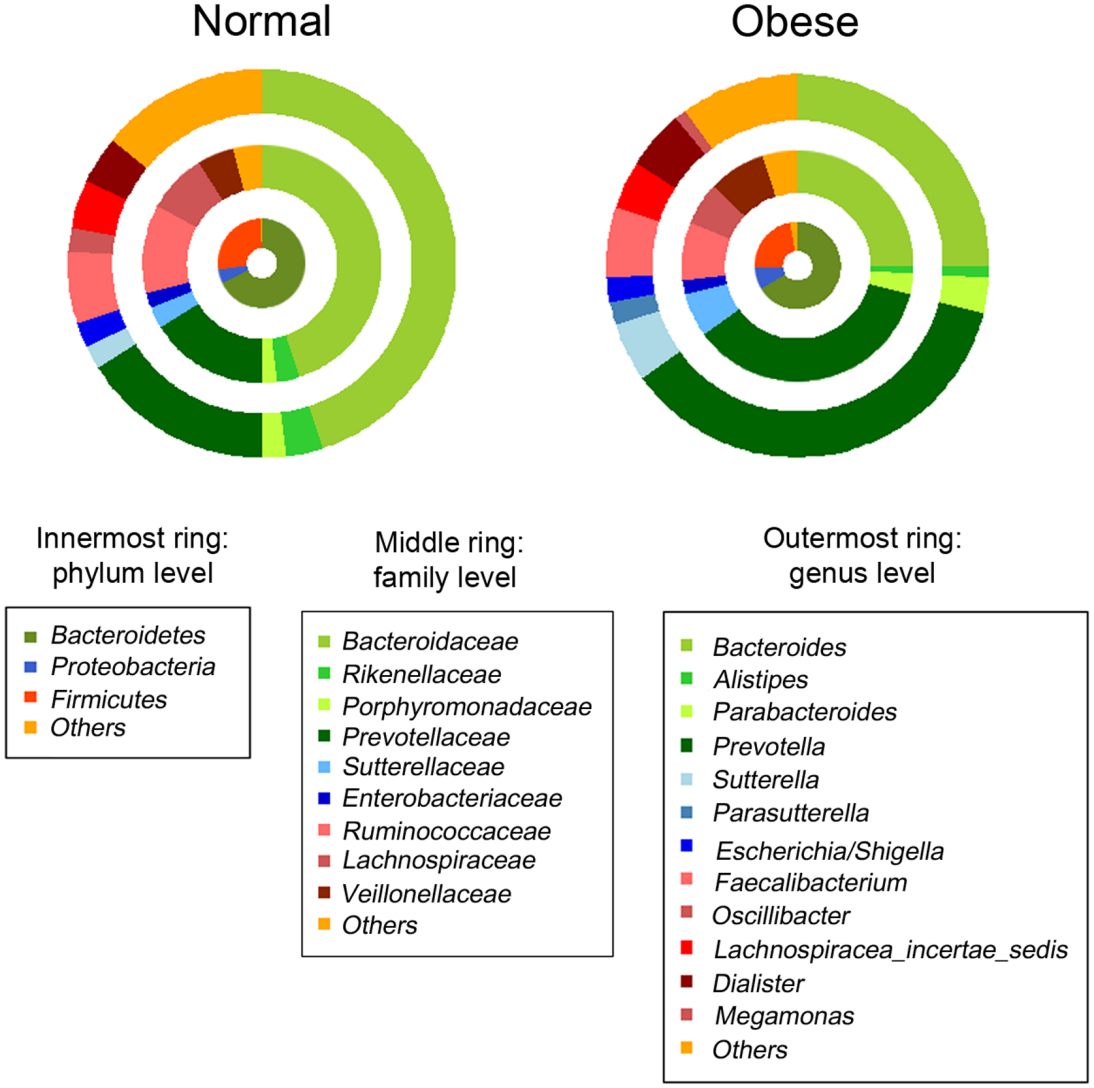
Average composition of the microbiota in fecal samples from normal and obese adolescents according to taxonomic rank. The innermost ring of the donut plots for both normal and obese individuals shows the composition at the phylum level, whereas the middle and outermost rings show the composition at the family and genus levels, respectively. There was a marked difference in the average proportions of *Bacteroides* and *Prevotella* between normal and obese samples at the genus level. This trend persisted at the family level. However, there were no significant differences in the *Bacteroidetes*, *Firmicutes*, and *Proteobacteria* populations in samples from normal and obese adolescents at the phylum level. All microbiota representing > 1% (mean value) composition are shown.

**Fig 2:**
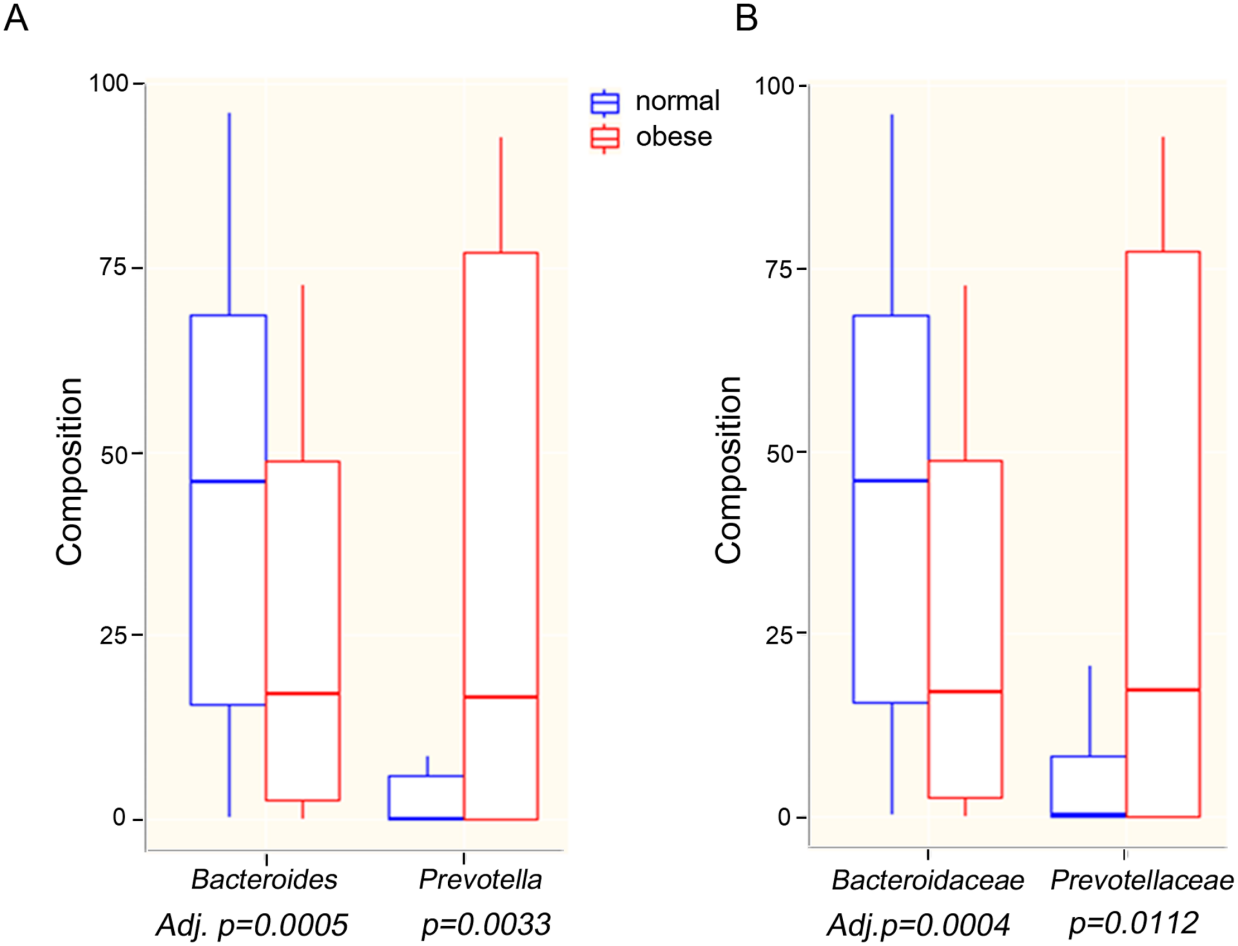
Mann-Whitney U test result showing differentially abundant microbiota in obese and normal adolescents. A. Genus level and B. Family level.

**Fig 3:**
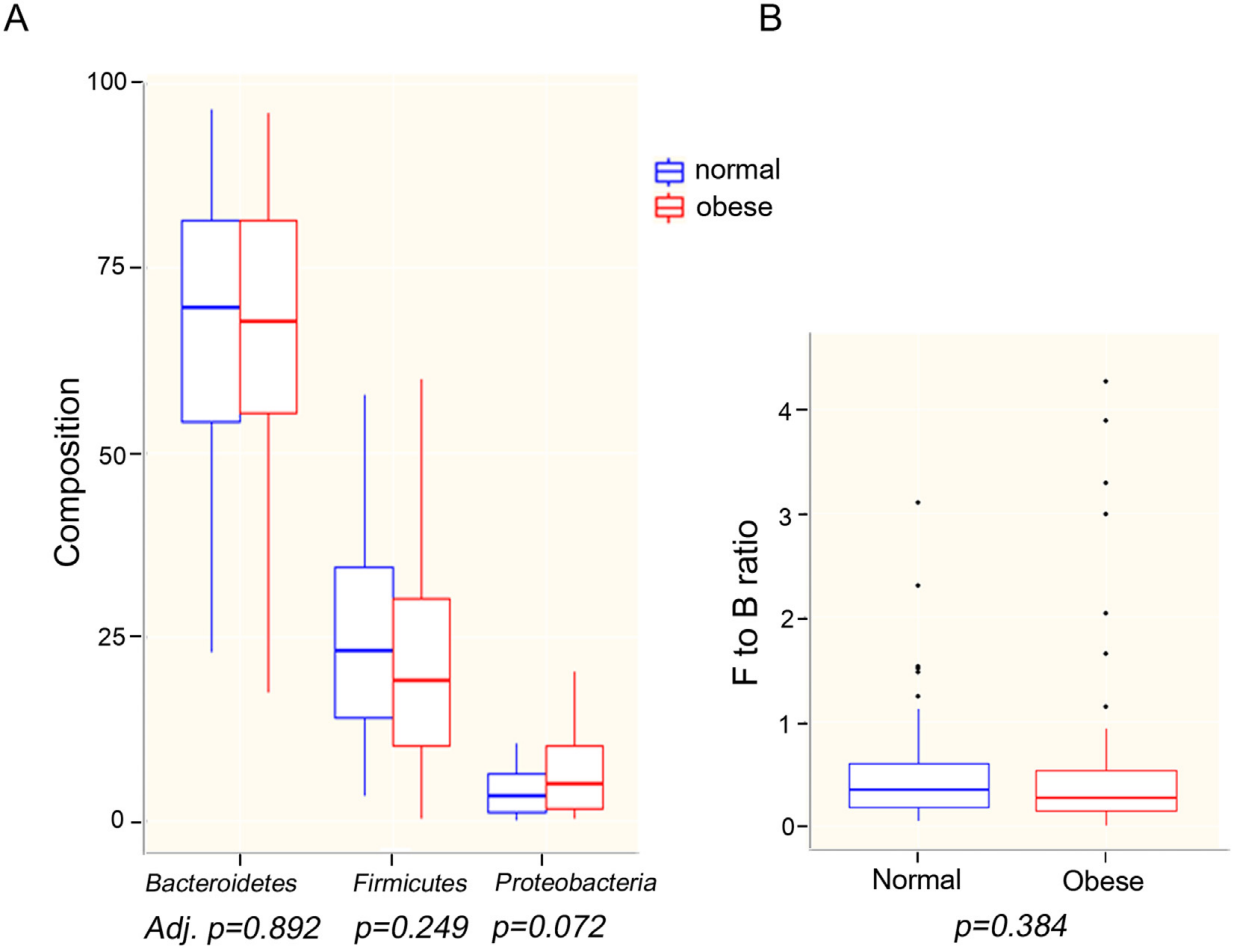
Mann-Whitney U test results at the phylum level. A. Box plots showing similar abundance of the *Bacteroidetes*, *Firmicutes*, and *Proteobacteria* populations in normal and obese adolescents. B. The *Firmicutes*-to-*Bacteroidetes* ratio in the gut of normal and obese adolescents. There was no significant difference in F/B ratio between normal and obese adolescents.

Fig 1 shows the average composition of the fecal microbiota from normal and obese adolescents according to taxonomic rank. The innermost ring of the donut plots for both normal and obese individuals shows the composition at the phylum level, whereas the middle and outermost rings show the composition at the family and genus levels, respectively. Notably, there was a marked difference in the average proportions of *Bacteroides* and *Prevotella* between normal and obese samples at the genus level. The proportion of *Bacteroides* was highest in normal children (45%), whereas that in obese adolescents was 25%. Conversely, the proportion of *Prevotella* in normal adolescents was 16%; however, it was highest in obese adolescents (35%). This trend persisted at the family level (from 45% to 25% and from 16% to 36% in normal and obese adolescents, respectively). The box plots in Fig 2 clearly show the differential abundance of these two bacterial taxa in obese and normal adolescents; however, this difference was not apparent at the phylum level, since both *Bacteroides* and *Prevotella* belong to the phylum *Bacteroidetes*. At the phylum level, we found no significant differences between the *Bacteroidetes*, *Firmicutes*, and *Proteobacteria* populations in normal and obese adolescents (Fig 3A). At this level, the microbial composition of samples from normal adolescents was similar to that observed in a study examining the gut microbiota of 20 Korean individuals [16]. The authors of that study reported that *Bacteroidetes* and *Firmicutes* were the two major microbial taxa, amounting to an average of 94.8% of sequence reads. In the present study, these taxa accounted for 94% (on average) of the sequence reads obtained from normal adolescents.

We next examined the F/B ratio, whose association with human obesity is unclear [17–19]. We found no significant difference in the F/B ratio between normal and obese adolescents (F/B ratio ± SD: F/B normal = 0.50 ± 0.53; F/B obese = 0.56 ± 0.86; *p* = 0.384) (Fig 3B).

The non-parametric Mann-Whitney U test also identified other bacterial taxa that differed significantly in terms of composition between obese and normal samples. We found significant differences in the *Alistipes*, *Faecalibacterium*, and *Oscillibacter* populations at the genus level (FDR-adjusted *p* = 0.0125, 0.0420, and 0.0007, respectively) (Table 2), and in the *Rikenellaceae*, *Sutterellaceae*, *Ruminococcaceae*, and *Veillonellaceae* populations at the family level (FDR-adjusted *p* = 0.0112, 0.0450, 0.0022, and 0.0450, respectively) (Table 3).

**Table 2:** List of taxa showing different abundance between obese and normal samples at the genus level.

Genus	P-value* (unadjusted)	P-value (FDR-adjusted)	Obese (median)	Normal (median)	Fold change (Obese/Normal)
*Bacteroides*	4.80E-05	**0.0005** ^†^	16.956	45.836	0.37
*Alistipes*	0.0050	**0.0125**	0.292	1.081	0.27
*Parabacteroides*	0.2150	0.2688	0.443	0.840	0.53
*Prevotella*	0.0010	**0.0033**	16.517	0.017	983.43
*Sutterella*	0.1240	0.2067	1.434	0.422	3.40
*Escherichia_Shigella*	0.9660	0.9660	0.030	0.026	1.15
*Faecalibacterium*	0.0210	**0.0420**	1.639	3.038	0.54
*Oscillibacter*	1.48E-4	**0.0007**	0.167	0.502	0.33
*Lachnospiracea_incertae_sedis*	0.1510	0.2157	2.100	2.786	0.75
*Dialister*	0.2970	0.3300	1.957	0.422	4.64

*Mann-Whitney U test.

^†^FDR-adjusted significant *p*-values are marked in bold.

**Table 3:** List of taxa showing different abundance between obese and normal samples at the family level.

Family	P-value* (unadjusted)	P-value (FDR-adjusted)	Obese (median)	Normal (median)	Fold change (Obese/Normal)
*Bacteroidaceae*	4.80E-05	**0.0004** ^†^	16.956	45.836	0.37
*Rikenellaceae*	0.0050	**0.0112**	0.292	1.081	0.27
*Porphyromonadaceae*	0.1370	0.1541	0.586	1.120	0.52
*Prevotellaceae*	0.0040	**0.0112**	17.317	0.084	206.92
*Sutterellaceae*	0.0280	**0.0450**	3.009	1.522	1.98
*Enterobacteriaceae*	0.4440	0.4440	0.051	0.033	1.53
*Ruminococcaceae*	4.98E-4	**0.0022**	3.820	9.450	0.40
*Lachnospiraceae*	0.0630	0.0810	4.197	5.780	0.73
*Veillonellaceae*	0.0300	**0.0450**	3.463	2.233	1.55

*Mann-Whitney U test.

^†^FDR-adjusted significant *p*-values are marked in bold.

### Association between BMI and biochemical markers

We next measured the correlation between taxa at the genus level (based on the five taxa showing statistically significant differences in composition between obese and normal adolescents at the genus level) and the BMI z-score or levels of biochemical markers (Table 4). Consistent with previous observations, we found that the *Bacteroides* and *Prevotella* populations were significantly associated with the BMI z-score (p < 0.0001 and 0.0001, respectively); the former showed a negative correlation while the latter showed a positive correlation. We also found that *Alistipes* was negatively correlated with BMI, although the correlation was less significant (*p* = 0.0360).

**Table 4:** Association between the composition of the gut microbiota and body mass index (BMI) z-score and biochemical markers according to *Pearson*'s *r* (*p*-value).

	BMI zscore*	Glucose	TG	Tchol	HDLc	hs-crp
*Bacteroides*	-0.37	-0.06	-0.25	-0.27	0.21	-0.25
	(**<0.0001**)^**†**^	(0.5195)	(**0.0049**)	(**0.0023**)	(**0.0165**)	(**0.0038**)
*Prevotella*	0.32	0.14	0.14	0.16	-0.10	0.21
	(**0.0001**)	(0.1107)	(**0.0394**)	(0.0765)	(0.2545)	(**0.0150**)
*Alistipes*	-0.18	0.01	-0.15	-0.14	0.03	-0.04
	(**0.0360**)	(0.8739)	(0.0891)	(0.1070)	(0.7336)	(0.6719)
*Faecalibacterium*	-0.106	-0.12	-0.11	-0.03	0.03	-0.05
	(0.2242)	(0.1676)	(0.2077)	(0.7477)	(0.7457)	(0.5392)
*Oscillibacter*	-0.16	-0.06	-0.07	0.08	0.10	-0.18
	(0.0644)	(0.5025)	(0.4056)	(0.3534)	(0.2603)	(**0.0360**)

*BMI z-score = Z-score-converted value from the 2007 Korean growth chart.

Blood data (Glucose, triglycerides (TG), total cholesterol (Tchol), high-density lipoprotein cholesterol (HDLc) and high-sensitive C-reactive protein (hs-crp)) were log-transformed prior to analysis. Tested by age- and gender-adjusted partial correlation analysis.

^†^Significant associations (value < 0.05) are marked in bold.

TG, Tchol, and hs-crp showed a negative correlation with the proportion of *Bacteroides* (*p* = 0.0049, 0.0023, and 0.0038, respectively), while HDLc showed a positive correlation (*p* = 0.0165). By contrast, TG and hs-crp showed a positive correlation with the *Prevotella* population (*p* = 0.0394 and 0.0150, respectively). The *Oscillibacter* population showed a negative correlation with hs-crp, although with less significance (*p* = 0.0360).

### Association rule mining

A machine learning algorithm called association rule mining was used to identify patterns of association between multiple bacterial taxa in obese and normal adolescents [27]. Rules were generated based on the five taxa showing significantly different compositions in obese and normal adolescents at the genus level (Table 2). The composition of each bacterial taxon was categorized into four groups (1, 2, 3, or 4) according to the quartile values (see the applied category for rule generation in Table 5). Five “obese” rules and six “normal” rules, each with an accuracy ≥ 80%, are presented in Table 5. The most accurate rule is “normal” rule 6, which states that if the proportion of *Bacteroides* is in the 4th quartile and that of *Prevotella* in the 1st quartile, the sample is classified as “normal” (accuracy, 95%). Similarly, “normal” rule 7 states that if the proportions of both *Bacteroides* and *Oscillibacter* are in the 4th quartile, then the sample is classified as “normal” (accuracy, 89%). “Obese” rule 1 states that if the proportion of *Prevotella* is in the 4th quartile and that of both *Faecalibacterium* and *Oscillibacter* is in the 1st quartile, then the sample is classified as “obese” (accuracy, 86%). Interestingly, if we compare rule 5 with rule 11, we see that the proportion of *Oscillibacter* appears to account for all the differences between the obese and normal states. Namely, in both rules, the proportions of the first two genera (*Bacteroides* and *Faecalibacterium*) are in the same quartile. However, with the first quartile proportion of *Oscillibacter*, they became to the obese rule and with the fourth quartile proportion, to the normal rule. Indeed, except for rule 4, all obese rules in Table 5 were involved with the first quartile proportion of *Oscillibacter* whereas most normal rules (except for rule 9) were with the fourth quartile proportion.

**Table 5:** Association rules generated by association rule mining.

**Obese rules**					
(Rule 1) *Prevotella* = {4}, *Faecalibacterium* = {1}, *Oscillibacter* = {1} → Obese (0.86)					
(Rule 2) *Bacteroides* = {3}, *Oscillibacter* = {1} → Obese (0.86)					
(Rule 3) *Prevotella* = {2}, *Faecalibacterium* = {1}, *Oscillibacter* = {1} → Obese (0.83)					
(Rule 4) *Bacteroides* = {1}, *Alistipes* = {1}, *Faecalibacterium* = {2} → Obese (0.80)					
(Rule 5) *Bacteroides* = {2}, *Faecalibacterium* = {1}, *Oscillibacter* = {1} → Obese (0.80)					
**Normal rules**					
(Rule 6) *Bacteroides* = {4}, *Prevotella* = {1} → Normal (0.95)					
(Rule 7) *Bacteroides* = {4}, *Oscillibacter* = {4} → Normal (0.89)					
(Rule 8) *Bacteroides* = {3}, *Faecalibacterium* = {4}, *Oscillibacter* = {4} → Normal (0.86)					
(Rule 9) *Bacteroides* = {4}, *Oscillibacter* = {2} → Normal (0.86)					
(Rule 10) *Bacteroides* = {3}, *Oscillibacter* = {4} → Normal (0.85)					
(Rule 11) *Bacteroides* = {2}, *Faecalibacterium* = {1}, *Oscillibacter* = {4} → Normal (0.80)					
**Rule explanation (Rule 1)**					
Original rule	*Prevotella* = {4}, *Faecalibacterium* = {1}, *Oscillibacter* = {1} → Obese (0.86)				
Rule explanation	If the compositions of *Prevotella* > 66, *Faecalibacterium* < 0.7 and *Oscillibacter* < 0.1; the sample is obese with 86% accuracy				
**Applied category for rule generation**					
Category	*Bacteroides*	*Alistipes*	*Prevotella*	*Faecalibacterium*	*Oscillibacter*
1	<4	<0.04	0	<0.7	<0.1
2	4–36	0.04–0.5	0–0.1	0.7–2	0.1–0.3
3	36–60	0.5–2	0.1–66	2–7	0.3–1
4	>60	>2	>66	>7	>1

## Discussion

Here, we examined fecal samples from 67 obese and 67 normal Korean adolescents to identify the association between the composition of the gut microbiota and childhood obesity. We used the QIAamp DNA Stool Kit to extract DNA because this kit shows high efficiency when used with different protocols [31, 32]. A study that compared mechanical and enzymatic methods of DNA extraction indicated that the mechanical cell disruption results in higher bacterial diversity and improves DNA extraction efficiency [33]. However, the microbiota was highly similar regardless of the extraction method used.

We found that the composition of the microbiota in samples from normal adolescents was in agreement with that reported in a study that examined the gut microbiota of 20 Korean individuals at the phylum level [16] and a study that examined the gut enterotypes in Korean monozygotic twins [34]. Even though two of the 20 Korean samples were from children, we can assume that normal Korean adolescents and adults have similar gut microbial compositions, at least at the phylum level. The results of the study of Korean monozygotic twins indicated that the microbiota of healthy Koreans clustered into two enterotypes, which are dominated by either *Bacteroides* or *Prevotella*. However, these enterotypes were not significantly correlated with biomarkers such as age, BMI, blood pressure, Tchol, or TG. Only one biomarker, serum uric acid, was different between the two enterotypes [34].

Many studies have attempted to identify an association between the composition of the gut microbiota and obesity [7–11, 35]. Some results are consistent whereas others are contradictory. For example, we found no significant difference in the F/B ratio between obese and normal adolescents, which is in line with a study by Karlsson et al., [17], who examined this ratio in obese and lean Swedish preschool children. By contrast, one Spanish study [19] and one Belgian study [18] reported an increased F/B ratio in obese children. Considering that the association between the composition of the gut microbiota and obesity appears to differ according to age and geographical location [13–16], we can surmise that the results of these studies may apply only to specific populations and to specific age groups. We assume that the inconsistencies are due to the complex relationships between genetic, environmental, technical, and/or clinical factors. Therefore, more integrative approaches will be needed if we are to fully understand this complex association.

Most studies searching for a link between the microbial composition in the gut and obesity focused on only one specific taxonomic rank when comparing normal and obese individuals. Here, we examined bacterial composition at the genus, family, and phylum levels. We found that the proportions of *Bacteroides* (*Bacteroidaceae*) and *Prevotella* (*Prevotellaceae*) were markedly different in normal and obese adolescents at both the genus and family levels. The adolescents examined in the present study were morbidly obese (BMI, 35.4± 2.9 kg/m^2^ or ≥ 99^th^ BMI percentile). Likewise, Zhang et al. [36] reported that *Prevotellaceae*, a subgroup of *Bacteroides*, were highly enriched in severely obese subjects. Here, we found that *Bacteroides* was the most prevalent genus in the normal adolescent group, a finding that is inconsistent with that of Agans et al., who found that *Ruminococcus* was the most prevalent genus in the normal adolescent group [37]. A metagenomic analysis examining the number of gut microbial genes, and thus the “richness” of the gut microbiota, indicated that obese individuals were more likely to possess low gene count (LGC) microbiota [20]. The significant difference in the *Faecalibacterium* proportion between obese and lean individuals in that study is consistent with the results reported herein. We believe that these adiposity- and age-specific differences in the bacterial populations are more informative, and will increase our understanding of the association between gut microbial composition and obesity.

We also found that the size of the *Bacteroides* population was negatively associated with TG, Tchol, and hs-crp levels (*p* = 0.0049, 0.0023, and 0.0038, respectively) but positively associated with HDLc (*p* = 0.0165); however, these results do not agree with those of Bervoets et al., who examined gut microbiota composition in 26 obese and 27 lean children aged 6–16 years [18]. They found no significant association between the *Bacteroides* population and biochemical markers such as glucose, HDLc, or Tchol; however, they did find a positive association between *Lactobacillus* and plasma hs-CRP levels (*p* = 0.007). It is not clear whether the difference between the results of their study and our own is due to the smaller sample sizes or to population differences.

We hypothesized that the populations of multiple bacterial taxa are associated with obesity; therefore, we attempted to identify patterns using association rule mining. By categorizing the composition of statistically significant microbiota, we devised several rules to determine whether a sample can be classified as “obese” or “normal”. We found that when *Bacteroides* and *Faecalibacterium* were equally abundant, the abundance of *Oscillibacter* was the major determinant of obese or normal status. Other studies have also associated the abundance of *Oscillibacter* with obesity. For example, Tims et al. examined twins that were discordant in terms of BMI status and found that *Oscillibacter* was more abundant in the leaner twin [38]. Walker et al. examined the effect of a precisely controlled diet in 14 overweight men; they found that *Oscillibacter* group increased on the resistant starch (RS) and a reduced carbohydrate weight loss (WL) diet [39]. Even though the authors were unsure whether *Oscillibacter* is a starch degrader, they assumed that the increase in the population must be due to the diet itself. Further studies are needed to verify this observation. To investigate what happens when we take into account genera other than the five showing significant differences in terms of population, we examined 28 other genera from both obese and normal samples whose median values for rule generation were greater than 0 (Table A in S1 File). As expected, the rule set was larger than that obtained for five genera; indeed, 12 “obese” rules and 17 “normal” rules, each with an accuracy ≥ 80%, were generated. However, including the rarer genera did not lead to an improvement in overall accuracy. One interesting finding is that the obese rules were often met when the proportion of *Sutterella* was in the 3rd or 4th quartiles. However, since these rules were generated from a relatively small sample set, they may not be generalizable. We believe that examining a larger sample set in the future will identify more reliable rules.

Recent studies examined the correlation between bacterial composition and diet. De Filippo et al. showed that the fecal communities in rural African children were different from those in European children [15]. African children, who consume a diet low in fat and protein and rich in plant-based foods, have a significantly enriched *Bacteroidete*s population and a depleted *Firmicutes* population when compared with European children. Other studies also demonstrate that the *Prevotella*-enriched enterotype is associated with a high carbohydrate diet [18, 40], while the *Bacteroides*-enriched enterotype is correlated with a diet high in protein and animal fat [41]. These results suggest that alterations in diet induce changes in the gut microbiota. Even though we could not evaluate the effects of dietary pattern and gut microbial composition on obesity in the current study, we believe that this should be taken into consideration in future in-depth studies. In addition, the present study was of cross-sectional design. Therefore, additional prospective studies are required to fully determine the causal relationships between gut microbiota, diet, and obesity.

## Supporting Information

S1 File **Figure A, Number of sequencing reads and operational taxonomic units (OTUs).** A. Number of reads per sample. B. Number of OTUs per sample. **Figure B, Alpha diversity (Shannon index) of the operational taxonomic units.** Shannon index for each sample. **Figure C, Beta diversity principal component analysis plot for normal and obese individuals. Table A, Association rules generated by association rule mining using 28 different genera.**
(PDF)
