# Correction: Obesity Alters the Microbial Community Profile in Korean Adolescents

**DOI:** 10.1371/journal.pone.0138015

**Published:** 2015-09-04

**Authors:** Hae-Jin Hu, Sin-Gi Park, Han Byul Jang, Min-Kyu Choi, Kyung-Hee Park, Jae Heon Kang, Sang Ick Park, Hye-Ja Lee, Seung-Hak Cho

The fourth author’s name is spelled incorrectly. The correct name is: Min-Kyu Choi. The correct citation is: Hu H-J, Park S-G, Jang HB, Choi M-K, Park K-H, Kang JH, et al. (2015) Obesity Alters the Microbial Community Profile in Korean Adolescents. PLoS ONE 10(7): e0134333. doi:10.1371/journal.pone.0134333

There is an error in affiliation 4 for author Min-Kyu Choi. Affiliation 4 should be: Department of Family Medicine, Kangnam Sacred Heart Hospital, Hallym University, Seoul, Republic of Korea
