# Supplementary material for: Obesity Alters the Microbial Community Profile in Korean Adolescents
Source: PLoS One. 2015 Jul 31;10(7):e0134333. doi: 10.1371/journal.pone.0134333 (PMC4521691; doi:10.1371/journal.pone.0134333)
Supplement: S1 File — Figure A, Number of sequencing reads and operational taxonomic units (OTUs). A. Number of reads per sample. B. Number of OTUs per sample. Figure B, Alpha diversity (Shannon index) of the operational taxonomic units. Shannon index for each sample. Figure C, Beta diversity principal component analysis plot for normal and obese individuals. Table A, Association rules generated by association rule mining using 28 different genera. (PDF) [file pone.0134333.s001.pdf]

S1 File: Figure A.

A

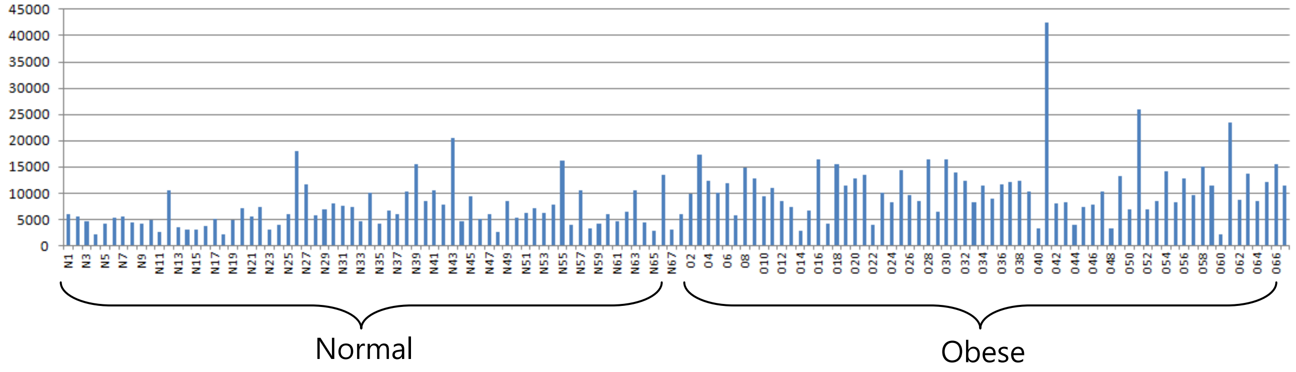

B

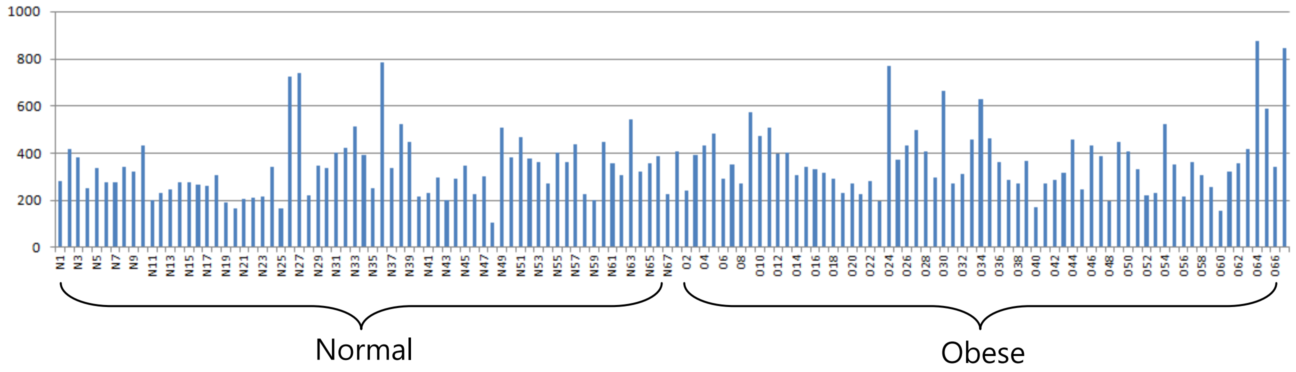

Figure B.

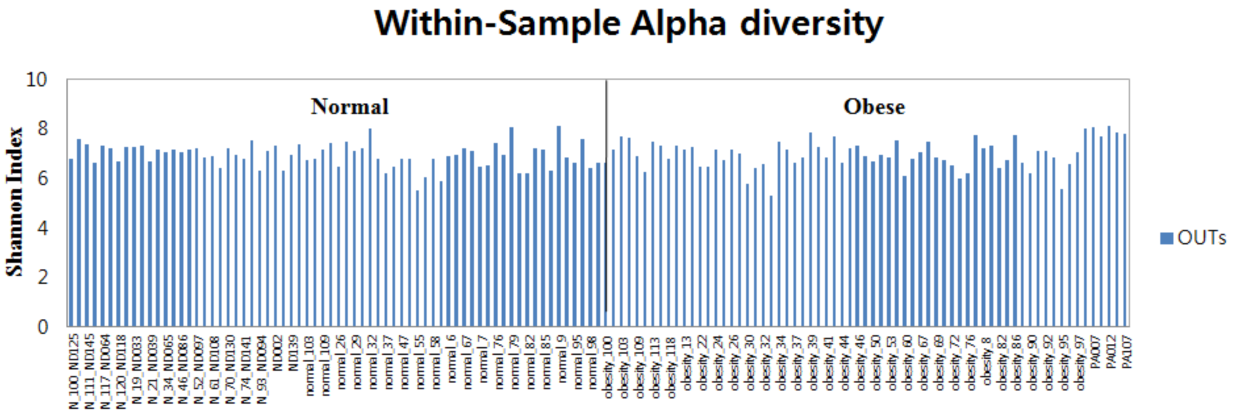
Within-Sample Alpha diversity
Shannon Index
10
8
6
4
2
0
Normal
Obese
OUTs

Figure C.

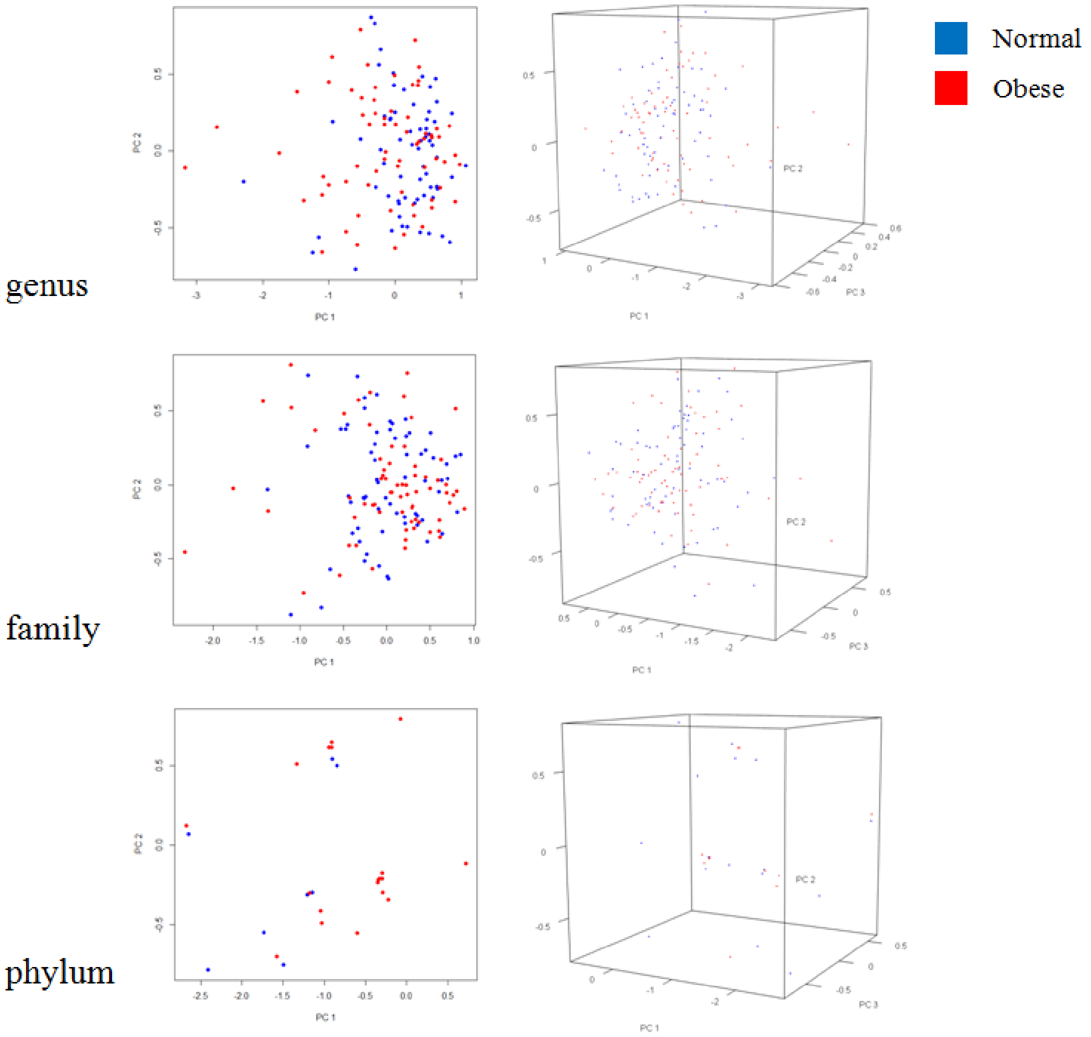

Table A.

Decoded Rules

---

(1) Bacteroides = {1}, Escherichia_Shigella = {3} -> Obese (0.90)

(2) Sutterella = {3}, Clostridium_XVIII = {2}, Bifidobacterium = {1} -> Obese (0.90)

(3) Bacteroides = {2}, Oscillibacter = {1}, Streptococcus = {2} -> Obese (0.89)

(4) Faecalibacterium = {1}, Sutterella = {4} -> Obese (0.89)

(5) Sutterella = {3}, Ruminococcus = {2}, Bifidobacterium = {1} -> Obese (0.89)

(6) Sutterella = {3}, Clostridium_XI = {2}, Bifidobacterium = {1} -> Obese (0.89)

(7) Bacteroides = {2}, Oscillibacter = {1}, Subdoligranulum = {1} -> Obese (0.88)

(8) Bacteroides = {1}, Dialister = {4} -> Obese (0.85)

(9) Bacteroides = {1}, Bifidobacterium = {2} -> Obese (0.85)

(10) Sutterella = {4}, Clostridium_XI = {2} -> Obese (0.82)

(11) Sutterella = {4}, Haemophilus = {2} -> Obese (0.81)

(12) Sutterella = {4}, Bifidobacterium = {4} -> Obese (0.80)

(13) Bacteroides = {4}, Prevotella = {1}, Escherichia_Shigella = {2} -> Normal (0.92)

(14) Bacteroides = {4}, Prevotella = {1}, Gemmiger = {2} -> Normal (0.92)

(15) Bacteroides = {4}, Prevotella = {1}, Ruminococcus = {2} -> Normal (0.91)

(16) Bacteroides = {4}, Prevotella = {1}, Clostridium_IV = {2} -> Normal (0.91)

(17) Bacteroides = {4}, Prevotella = {1}, Clostridium_XIVb = {2} -> Normal (0.91)

(18) Bacteroides = {4}, Prevotella = {1}, Streptococcus = {2} -> Normal (0.91)

(19) Oscillibacter = {4}, Clostridium_IV = {4}, Blautia = {3} -> Normal (0.91)

(20) Bacteroides = {4}, Flavonifractor = {3} -> Normal (0.91)

(21) Oscillibacter = {3}, Sutterella = {2} -> Normal (0.88)

(22) Bacteroides = {2}, Oscillibacter = {4}, Blautia = {3} -> Normal (0.88)

(23) Prevotella = {3}, Oscillibacter = {4}, Blautia = {3} -> Normal (0.88)

(24) Bacteroides = {3}, Faecalibacterium = {4}, Oscillibacter = {4} -> Normal (0.86)

(25) Bacteroides = {3}, Oscillibacter = {4}, Clostridium_IV = {4} -> Normal (0.86)

(26) Bacteroides = {3}, Oscillibacter = {4}, Blautia = {4} -> Normal (0.86)

(27) Bacteroides = {3}, Oscillibacter = {4}, Clostridium_XI = {2} -> Normal (0.86)

(28) Bacteroides = {3}, Oscillibacter = {4}, Escherichia_Shigella = {2} -> Normal (0.83)

(29) Bacteroides = {3}, Oscillibacter = {4}, Flavonifractor = {2} -> Normal (0.83)
